# Automatic Identification of Lung Opacities Due to COVID-19 from Chest X-ray Images—Focussing Attention on the Lungs

**DOI:** 10.3390/diagnostics13081381

**Published:** 2023-04-10

**Authors:** Julián D. Arias-Londoño, Álvaro Moure-Prado, Juan I. Godino-Llorente

**Affiliations:** ETSI Telecomunicación, Universidad Politécnica de Madrid, Avda. Ciudad Universitaria, 30, 28040 Madrid, Spain

**Keywords:** COVID-19, decision support system, chest X-ray, lesions detection, deep learning

## Abstract

Due to the primary affection of the respiratory system, COVID-19 leaves traces that are visible in plain chest X-ray images. This is why this imaging technique is typically used in the clinic for an initial evaluation of the patient’s degree of affection. However, individually studying every patient’s radiograph is time-consuming and requires highly skilled personnel. This is why automatic decision support systems capable of identifying those lesions due to COVID-19 are of practical interest, not only for alleviating the workload in the clinic environment but also for potentially detecting non-evident lung lesions. This article proposes an alternative approach to identify lung lesions associated with COVID-19 from plain chest X-ray images using deep learning techniques. The novelty of the method is based on an alternative pre-processing of the images that focuses attention on a certain region of interest by cropping the original image to the area of the lungs. The process simplifies training by removing irrelevant information, improving model precision, and making the decision more understandable. Using the FISABIO-RSNA COVID-19 Detection open data set, results report that the opacities due to COVID-19 can be detected with a Mean Average Precision with an IoU > 0.5 (mAP@50) of 0.59 following a semi-supervised training procedure and an ensemble of two architectures: RetinaNet and Cascade R-CNN. The results also suggest that cropping to the rectangular area occupied by the lungs improves the detection of existing lesions. A main methodological conclusion is also presented, suggesting the need to resize the available bounding boxes used to delineate the opacities. This process removes inaccuracies during the labelling procedure, leading to more accurate results. This procedure can be easily performed automatically after the cropping stage.

## 1. Introduction

COVID-19 is caused by the SARS-CoV-2 virus, and since its first appearance in Wuhan in December 2019, it has spread rapidly around the world with a reproduction number (R0) estimated between 1.4 and 2.4 in Europe [1], and between 2.24 and 3.58 in China [2].

Since its appearance, current estimates report that SARS-CoV-2 has infected more than 677 people, which is the current number of active cases at the time of writing approximately 20 M [3]. During this time, the virus has mutated several times, changing many aspects that had a small to huge impact on the properties of the virus, such as its spreading factor, its mortality, or the performance of vaccines.

SARS-CoV-2 belongs to the same family of viruses as SARS-CoV, which causes Severe Acute Respiratory Syndrome (SARS), and MERS-CoV, responsible for the Middle East Respiratory Syndrome (MERS). These types of viruses are known as coronaviruses, and their main characteristic is that they contain a single-stranded RNA enclosed in a capsid (the protein shell of a virus) with spikes, therefore, resembling the corona-like appearance of the virus. These coronaviruses share many similarities, such as their manifestations of increased viral pneumonia or their survival decay.

COVID-19 is a type of respiratory disease that is transmitted mainly through the air through respiratory droplets and aerosols from mucous membranes of about 25 µm in size. The incubation period of the virus is approximately 4 to 7 days from the initial exposure of the individual to the virus [4]. COVID-19 has been shown to be much more infectious, but has a lower mortality rate than SARS.

Symptoms related to COVID-19 disease are cough, fever, fatigue, headache, and muscle pain. The loss of sense of smell (anosmia) and the loss of taste (dysgeusia) have also been frequently reported among patients. Around 30% of infected people also experience shortness of breath (dyspnea), and 10% also report gastrointestinal problems. Additionally, 30% of patients usually suffer from cardiovascular complications, which can include a variety of cardiomyopathies [5]. However, the most serious complication of patients with COVID-19 is acute respiratory distress syndrome, which often leads to high hospitalisation and mortality rates experienced during the pandemic. Due to the primary affection of the respiratory system, COVID-19 leaves fibrosis traces that are visible on chest radiological images in the form of ground glass opacities [6,7].

### 1.1. Motivation

Plain chest radiography (CXR) and computer tomography (CT) are two radiological techniques that have been shown to have great clinical value in the diagnosis and evaluation of patients with COVID-19. These techniques are valuable in diagnosis, medical triage, and/or therapy. In [8], it was shown that these imaging techniques were shown to be useful in clinical scenarios during the pandemic. For patients with mild symptoms of COVID-19, radiological images also provide a baseline for evaluating the patient’s condition. In the case of patients with moderate to severe symptoms, it also helps to identify lung and heart abnormalities and allows a more informed medical decision. CXR is also a great aid for fast triage. Finally, chest radiological tests are also recommended if the patient worsens so that he can be treated accordingly. In any case, radiological findings are not used as the primary screening tool to detect COVID-19, but rather to determine its severity and potential complications [9].

**Figure 1:**
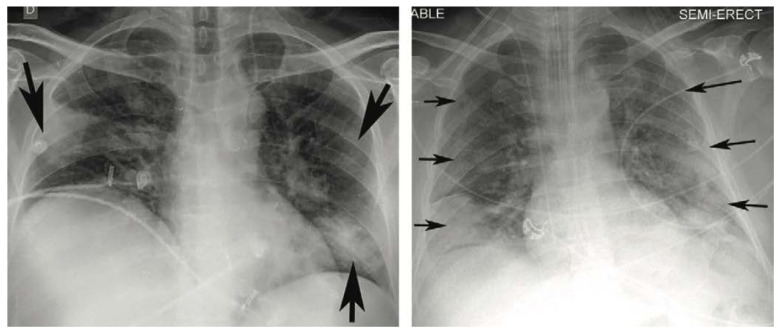
Typical appearances of COVID-19 in the form of lung opacities. CXR images obtained from the RICORD data set [10]. Arrows pointing to the opacities.

Many investigations report a variety of appearances in CXR of COVID-19 patients such as: [8,10,11,12,13]. The most typical reported appearances are multifocal bilateral and peripheral lung opacities, usually round-shaped and predominant in the lower part of the lungs [14], although there might be other appearances, such as central or upper lung opacities, and also pneumothoraxes, pleural effusions, pulmonary oedemas, lobar consolidations, lung nodules, and cavities [14]. In the same study, a classification of the type of appearance is made into four categories: typical appearance, atypical appearance, indeterminate appearance, and negative for pneumonia, depending on the type and location of the appearance. Figure 1 presents two examples of CXR images showing typical appearances in the form of lung opacities.

CXR images have been shown to be less sensitive to COVID-19 manifestations than those obtained with CT. In fact, one of the main limitations of using CXR as a diagnostic tool is the absence of appearances in the lungs in the early stages of the disease and, in certain cases, throughout the course of the disease. In addition to this, breast size, poor positioning, and lack of patient inspiration can introduce artefacts in CXR, often leading to false positives. In general, the sensitivity of CXR in combination with a human expert has been estimated to be around 69%, while for CT is around 98% [15].

The individual study of the radiography of every patient is a difficult and time-consuming task, as it requires specialised personnel. Therefore, the design and implementation of automatic decision support systems (DSS) that can identify lesions due to COVID-19 are of interest not only to alleviate the workload of sanitation personnel, but also to possibly detect hard-to-find lung lesions that could be missed in a first rushed look at a CXR.

### 1.2. State of the Art

Since the pandemic began, much research has been carried out on using methods based on artificial intelligence (AI) to develop DSS for screening and evaluating COVID-19 using CXR images. In this context, the area of research that has received more attention in the literature is the design of automatic screening tools to identify the patient’s condition. Work has also been done to identify and segment associated lung lesions, but in a smaller number and mostly using CT as the main data source, leaving CXR in the second plane. The following review of the state-of-the-art focuses on work using artificial intelligence techniques to identify COVID-19 lesions from CXR images.

Most approaches developed previously to identify COVID-19-related lung lesions from CXR images were proposed in the context of the SIIM-FISABIO-RSNA COVID-19 Detection Challenge [16]. The challenge’s goal was twofold, including lesion detection and classification into four categories. Participants trained their models using the SIIM-FISABIO-RSNA COVID-19 Detection data set [17], which contains 6334 CXR images. The data set was manually annotated by experts with the corresponding bounding boxes for the lesions observed in the images.

The winner of the challenge [18] used an ensemble of four models (YoloV5-x6, EfficientDet D7, Faster Region-Based Convolutional Neural Network Feature Pyramid Network [Faster-R-CNN-FPN] with a ResNet-101 backbone, and a Faster-R-CNN-FPN with a ResNet-200 backbone) fed with images of 768 × 768, 768 × 768, 1024 × 1024, and 768 × 768 pixels, respectively. Models were initially trained to predict pneumonia bounding boxes using an external data set. Then the pre-trained models were further trained with the data set provided by the challenge. Strong data augmentation along with Test Time Augmentation (TTA) were used. In this solution, the models were used to extract pseudo-labels that predict the bounding boxes in the test set and in three additional COVID-19 data sets (PadChest, Pneumothorax, and VinDr-CXR). Those boxes with a confidence greater than 0.7 were accepted as pseudo-labels. Finally, all labels were used for the final training of the models. The obtained Mean Average Precision (mAP) with an Intersection over Union (IoU) > 0.5 (mAP@50) for the four mentioned models was 0.580, 0.590, 0.592, and 0.596, respectively. The mAP@50 of the ensemble was not reported. The solution that scored second in the challenge [18] used a set of YoloV5, YoloX, and EfficientDet. The images were resized to 384 × 384 and 512 × 512, respectively, and the mAP@50 of the ensemble reached 0.59. The solution in the third place [18] used EfficientDet D7X, EfficientDet D6 and SWIN RepPoints. The models were pre-trained on an external pneumonia data set using five folds. To improve their results, the authors used the predictions of their classification model to complement the training/validation data set. No results in terms of mAP were provided for this solution. In fourth place, [18] used a variation of YoloV5 grounded on a transformer backbone, although the authors did not give the implementation details. This model was ensembled with YoloV5-x6 and YoloV3 models and trained using images of size 512 × 512. The models were initially pre-trained on an external pneumonia data set, and to improve their results, the authors applied a post-processing technique based on the geometric mean to re-rank their bounding boxes scores. The reported mAP@50 was 0.562. In fifth place, ref. [18] proposes an ensemble of five models (EfficientDet D3, EfficientDet D5, YoloV5-l, YoloV5-x, and RetinaNet-101), which was built and combined with the results of an additional classification model trained for binary predictions between the classes ‘none’ and ‘COVID-19’. No mAP@50 was reported for this solution. In sixth place, ref. [18] used a Faster-R-CNN-FPN with an EfficientNet B7 backbone, which was trained using images of 800 × 800 pixels. The model was initially pre-trained with an external pneumonia data set. The authors used several training techniques: stochastic weight averaging, sharpness-aware minimisation, attentional-guided context FPN, and fixed feature attention using the feature pyramid from their classification model into the detection model for attention. Their solution achieved a validation mAP@50 of 0.585. The solution in the seventh place [18] used an ensemble of 3 models (DetectoRS50, UniverseNet50, UniverseNet101) trained using pseudo-labelled images with 800 × 800 pixels. The models were ensembled using the non-Maximun Weighted (NMW) technique instead of the Weighted Box Fusion (WBF) [19] used by all other solutions. The obtained mAP@50 was 0.553. In eighth place [18], the solution used an ensemble of 3 YoloV5 models (YoloV5-x6 with input size of 640 × 640, YoloV5 with input size 1280 × 1280, and YoloV5x with input size 512 × 512), along with a Cascade R-CNN model using inputs of 640 × 640. In this case, background images were used to reduce the false positive rate. No performance metrics were reported. In the ninth place solution [18], eight different models were grouped: four variations of EfficientDet (EfficientDet D0, EfficientDet D0, EfficientDet D3, EfficientDet Q2) and four variations of YoloV5 (YoloV5-s, YoloV5-m, YoloV5-x, 2-class YoloV5-s). The authors did not report metrics. In the tenth place, ref. [18] used an ensemble of YoloV5 and EfficientDet. To improve training, the authors used an alternative data set (the RICORD COVID-19 data set [10]) to create pseudo-labels. No validation metrics were reported for this solution.

In addition to the approaches of the SIIM-FISABIO-RSNA COVID-19 Detection Challenge, in [20] authors developed an ensemble of YoloV5 and EfficientNet to locate ground glass opacities from radiological images also obtained from the SIIM-FISABIO-RSNA COVID-19 Detection data set. No image pre-processing was used, and mosaic data enhancement was used to train the YoloV5 model. The images were resized to 512 × 512, and the results reported 0.6 as mAP@50, without cross-validation. In addition, in [21], a YoloV5 and a RetinaNet-101 model were trained with the same data set used in previous works, and ensembled using a WBF algorithm, resizing all images to 1333 × 800. The obtained mAP@50 was 0.552 after stratified cross-validation of K. The authors in [22] ensembled a YoloV5 model with YoloV5m and YoloV5x backbones, a YoloX model with YoloX-m and YoloX-d backbones, and an EfficientDet B5 net to detect lung opacities in CXR images of 512 × 512 pixels. Histogram equalisation was used as a preprocessing technique, and strong data augmentation was performed for the YoloV5 model using mosaic augmentation, HSV (for hue, saturation, lightness) scaling, random shearing, and rotation; image mixup, random scaling, shearing, and rotation for the YoloX; and brightness, contrast, shearing, scaling and cropping for the Efficientdet B5. The ensemble of these three models reported mAP@50 of 0.62 after a stratified cross-validation of K times. In [23] YoloV5 was used with the YoloV5s, YoloV5m, YoloV5l and YoloV5x backbones. The images were converted to.jpeg format, and no cross-validation was used. The best results reported 57% true positives and 48% false negative rate.

Table 1 presents a summary of the main characteristics of previous works reported in the state-of-the-art for the automatic identification of COVID-19 lesions using deep learning techniques along with their main characteristics. A common characteristic of all existing works is the use of the SIIM-FISABIO-RSNA COVID-19 Detection data set. This is the largest open data set currently available, and its use makes comparing results easier by establishing a common framework. However, comparing the results using the architectures that participated in the challenge with later proposals is not straightforward since, for the challenge, performance results were calculated using a test data set with labels that were not made open by the organisers. This means that the available data do not allow for a direct comparison of the results provided with the new architectural proposals. Furthermore, most of the proposed solutions to the challenge do not provide detailed information about their implementation, resulting in lack of reproducibility.

However, the remaining works reported in the state-of-the-art used all available 6334 images from the SIIM-FISABIO-RSNA COVID-19 detection data set. However, the data set contains a large number of studies with several images per patient, and even some of them are identical. It means that a fair comparison would require a common inclusion criterion before properly comparing the results. Furthermore, two of these schemes were tested without cross-validation, which could bias the results towards optimistic values.

Following the pre-processing methodology proposed in [24] for COID-19 detection, this article explores the effect of cropping radiological images in the lungs area and the semantic segmentation of the regions of interest (that is, the lungs area) on the identification of the bounding boxes containing the associated lesions. The goal is to guide the attention of the model by reducing the search space and also reducing the distracting artefacts that could confuse the networks. For this purpose, the paper evaluates four different off-the-shelf artificial neural networks (ANN) commonly used for object detection purposes. Two two-stage detectors: Faster R-CNN, Cascade R-CNN; and two one-stage detectors: RetinaNet, and YoloV5. In addition, different combinations of ensembles of these models were also tested according to common strategies in the state-of-the-art. The selection of these simple architectures aims to promote manageable designs that could be trained and deployed easier in medical practice and whose generalisation capabilities are also easier to evaluate, although similar pre-processing strategies can also be incorporated into more complex architectures.

This article also analyses the effect of image cropping and semantic segmentation of the lungs on estimating pseudo-labels to carry out a co-training-based [25,26] semi-supervised strategy, also following similar methods to those used in the best works presented at the FISABIO-RSNA COVID-19 Detection Challenge [16]. As noted above, the state-of-the-art typically uses an arbitrary confidence score of 0.7 as a threshold to decide whether to accept the estimated boxes as pseudo-labels or not [18]. However, the use of pseudo-labels have several drawbacks. First, other works have pointed out that the scores provided by modern ANNs are poorly calibrated [27]; therefore, the scores obtained are difficult to compare, so applying the same threshold to two models with similar accuracies can perform quite differently in detecting the bounding boxes. Second, the scores provided by object detectors are related to the estimation of the category associated with the detected object, but many previous works have shown that the classification scores are not strongly correlated with the precision of box localisation (see [28] and references therein). To evaluate this potential issue, this article presents two different experiments designed to evaluate the training using pseudo-labels for lung lesion detection, considering possible underconfident and overconfident models.

**Table 1:** Summary of the literature available in the field.

Reference	Model Architecture	Image Size	Pretrained	mAP@50	Corpus	Remarks
[29]	YoloV5, EfficientNet	512 × 512	No	0.600	SIIM-FISABIO-RSNA (only the training subset)	No crossvalidation
[21]	YoloV5, RetinaNet-101	1333 × 800	No	0.552	SIIM-FISABIO-RSNA (only the training subset)	–
[22]	YoloV5, YoloX, EfficietNet-B5	384 × 384, 512 × 512	No	0.620	SIIM-FISABIO-RSNA (only the training subset)	–
[23]	CNN-N	256 × 256, 512 × 512	No	–	SIIM-FISABIO-RSNA (only the training subset)	False Positive = 0.49, False Negative = 0.51, No crossvalidation
[18] 1st	YoloV5-x6, EfficientDet D7, Faster R-CNN FPN	768 × 768, 1024 × 1024	Yes	0.596	SIIM-FISABIO-RSNA	Pseudo labels
[18] 2nd	YoloV5, YoloX, EfficientDet D5	384 × 384, 512 × 512	Yes	0.590	SIIM-FISABIO-RSNA	YoloV5 with 5 different backbones
[18] 3th	EfficientDet D7X, EfficientDet D6, SWN-RepPoints/EfficientNet	640 × 640	Yes	Not provided	SIIM-FISABIO-RSNA	–
[18] 4th	YoloV5-transformer, YoloV5-x6, YoloV3	512 × 512	Yes	0.562	SIIM-FISABIO-RSNA	Geometric mean confidence postprocessing
[18] 5th	EfficientDet D3, EfficientDet D5, YoloV5-l, YoloV5-x, RetinaNet-101	512 × 512, 1333 × 800	Yes	Not provided	SIIM-FISABIO-RSNA	–
[18] 6th	Faster R-CNN, EfficientNet B7	800 × 900	Yes	0.585	SIIM-FISABIO-RSNA	–
[18] 7th	DetectoRS50, UniverseNet50, UniverseNet101	800 × 800	No	0.553	SIIM-FISABIO-RSNA	Pseudo-labels, NMW
[18] 8th	YoloV5-x6, YoloV5-x, Cascade R-CNN	640 × 640, 1280 × 1280	No	0.540	SIIM-FISABIO-RSNA	–
[18] 9th	EfficientDet D0,D3,Q2, YoloV5-s,m,x,2-classs	512 × 512, 640 × 640, 768 × 768	No	Not provided	SIIM-FISABIO-RSNA	–
[18] 10th	YoloV5, EfficientDet	Not provided	Not provided	0.620	SIIM-FISABIO-RSNA	Pseudo labels

Given the aforementioned, it is worth noting that this work is neither focussed on the development of new architectures nor on improving the accuracy of the state-of-the-art methods, but on discussing more interpretable approaches, and on identifying best methodological approaches and good practices.

The rest of the paper is organised as follows. Section 2 introduces the material and methods used in this paper. Section 3 mainly describes the results and an analysis of the different experiments. Section 4 ends with a discussion and conclusions.

## 2. Materials and Methods

This section presents the corpus used, the techniques developed for pre-processing the images, and the methods used for object detection.

### 2.1. Materials

This section describes the two data sets used for the training and validation of the models. These data sets and their respective annotations are freely available to all researchers for academic and non-commercial use.

#### 2.1.1. FISABIO-RSNA COVID-19 Detection Data Set

The models developed in this work were trained using the SIIM-FISABIO-RSNA COVID-19 Detection data set [17], which is also the main corpus used recurrently in the state-of-the-art. The data set was compiled from two public sources: BIMCV [30] and MIDRC-RICORD [10]. All medical imaging data and metadata were properly de-identified.

This corpus is built around the study levels and the image levels. The first term is used to identify each patient in the data set in the form of a complete study, since one study may contain one or more image levels (i.e., 1:N relationship), leading each level to different CXR images. The total number of studies is 6054, and the number of CXR 6334. Only 4294 (68%) had boxes that identified lung lesions. The remaining radiographs (without bounding boxes) are considered to belong to the control group ‘negative for pneumonia’. The corpus mixes anterior-posterior (AP) and posterior-anterior (PA) views obtained from both digital radiography (DX) and computed radiography (CR) devices. The number of cases exhibiting each of these typologies was not annotated.

**Figure 2:**
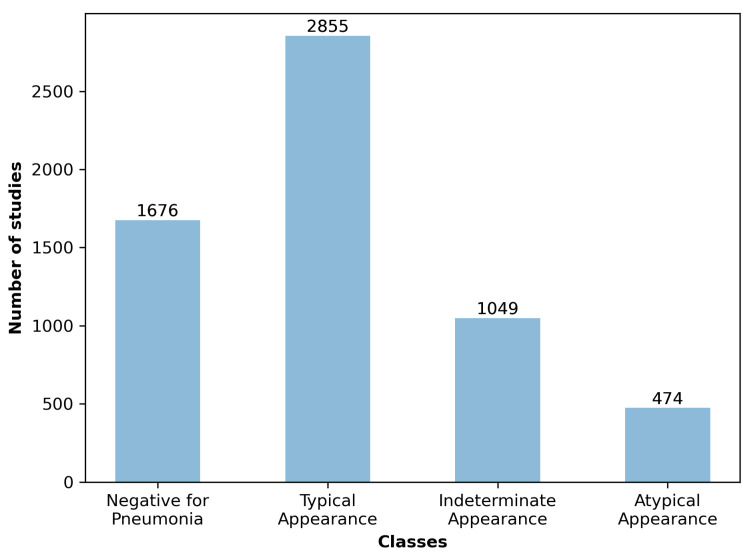
Study-level classes in the data set.

22 experts carried out the annotations: 13/22 were non-thoracic radiologists and 9/22 were thoracic sub-speciality radiologists. Every image was annotated only by a single radiologist out of 22. The experts received specific training and evaluation to agree on the criteria used to label the data set [17]. To assess the consistency of the evaluation, a 25 CXR test case was performed, obtaining a median percentage agreement of 86% between experts. Chest radiographs were annotated in four mutually exclusive categories, including ‘typical’, ‘indeterminate’, and ‘atypical appearance’ for COVID-19, or ‘negative for pneumonia’ (Figure 2). Bounding boxes were drawn over pulmonary opacities, and in those cases where two or more opacities were close to each other, an encompassing bounding box was drawn instead of several smaller boxes. No bounding boxes were placed on pleural effusions, masses/nodules, or pneumothoraces. No bounding boxes were placed for the ‘negative for pneumonia’ category.

**Figure 3:**
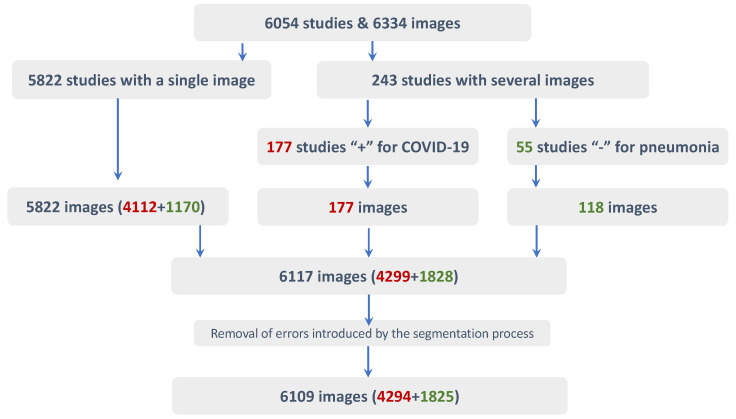
Schema representing the process followed to identify the samples used in the data set. In red, the number of COVID-19 positive samples (labelled with their corresponding bounding boxes). In green, the negative for pneumonia samples (without bounding boxes).

The final subset used for training is composed of 6117 images out of the 6334. 5822 of these samples were chosen from those studies with only one radiography, whether they have bounding boxes or not. Of the 177 studies with more than one CRX and only one image containing bounding boxes (i.e., ‘positive for COVID-19’), the one labelled was added to the subset (discarding 217 samples). And of the 55 studies with several CRX, but all labelled with no bounding boxes (i.e., ‘negative for pneumonia’), 118 images were added to the subset. Finally, 8 images out of 6117 were removed due to errors during the automatic lung segmentation process (1 out of 5822, and 1 out of 118). This process is schematically represented in Figure 3.

#### 2.1.2. HM Hospitales COVID-19 Data Set

In addition to the data set described above, the HM Hospitales COVID-19 data set was also used to apply a semi-supervised learning scheme based on a pseudo-labels strategy. This data set was compiled by HM Hospitals [31]. It contains all available clinical information on anonymous patients with the SARS-CoV-2 virus treated in different centres belonging to this company since the beginning of the pandemic in Madrid, Spain.

The corpus contains anonymised records of 2310 patients and includes several radiological studies for each patient corresponding to different stages of the disease. A total of 5560 CRX images are available in the data set, with an average of 2.4 image studies per subject, often taken in intervals of two or more days. Only patients with at least one positive PCR test or positive immunological test for SARS-CoV-2 were included in the study.

### 2.2. Methods

**Figure 4:**
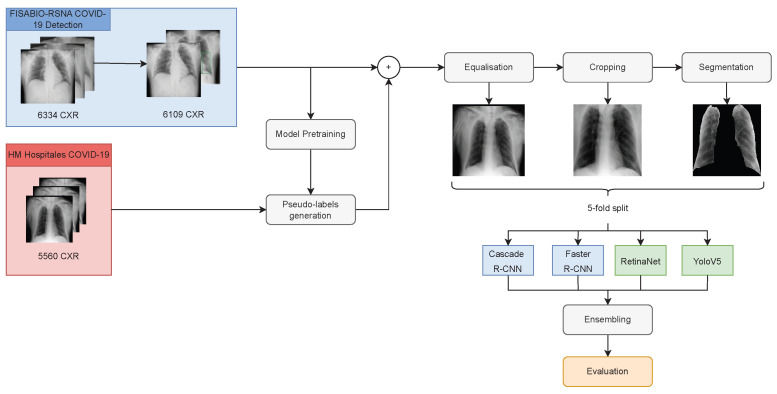
Schematic view of the procedure followed.

This section presents the pre-processing and data augmentation methods applied, the architectures used for modelling and their ensembling, and the semi-supervised approach followed for comparison purposes. Figure 4 presents a schematic view of the entire procedure carried out throughout this work.

#### 2.2.1. Pre-Processing

CXR images were converted to uncompressed greyscale ‘.png’ files, encoded with 8 bits, and pre-processed using DICOM *WindowCenter* and *WindowWidth* details (when needed). All images were converted to a *Monochrome 2* photometric interpretation.

**Figure 5:**
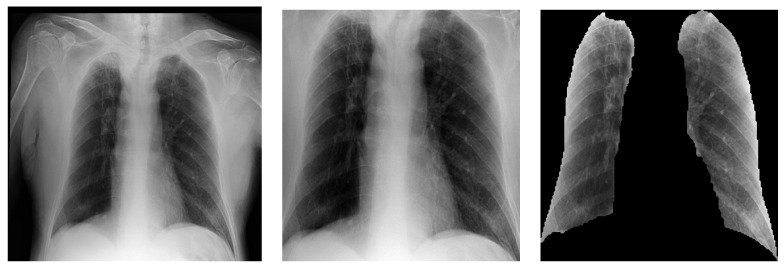
Examples of the three pre-processing schemes used. (**Left**): Raw image with equalization (taken from [17]). (**Centre**): Cropped image. (**Right**): Semantic segmentation of the lungs.

Pre-processing also includes resizing the image to 512 × 512 pixels. From this step on, three different pre-processing schemes, similar to those used in [24], were evaluated (Figure 5):

1. Image equalisation.
2. Semantic segmentation of the lungs + image equalisation.
3. Zooming and cropping to the rectangular region of interest (RoI) containing the lungs + image equalisation.

The first scheme processes the raw images, applying only an image equalisation using histogram equalisation and Contrast Limited Adaptive Histogram Equalisation (CLAHE) (Figure 5 Left). These two pre-processing tools are often used when working with radiological images since histogram equalisation causes the images to have a more uniform brightness distribution and CLAHE increases the contrast. The contrast obtained by CLAHE can be controlled by setting its clip limit. Typical values in the literature for this parameter for radiological images are 0.01 and 0.02. In this case, the clip limit was set to 0.02 (assuming a maximum value of 1). An amplitude normalisation of the image is also performed using typical values of the mean and standard deviation in the three RGB channels.

For the second scheme (Figure 5 Centre), the entire process is as follows:

1. The lungs were segmented from the original image using a U-Net semantic segmentation algorithm (Following the Keras implementation available at https://github.com/imlab-uiip/lung-segmentation-2d). The algorithm used reports *Intersection-Over-Union* (IoU) and Dice similarity coefficient scores of 0.971 and 0.985, respectively.
2. An external black mask is extracted to identify the external boundaries of the lungs.
3. The mask is dilated with a kernel of 5×5 pixels and is superimposed on the image.
4. Equalisation is performed considering only those pixels that take values different from zero.

The third scheme (Figure 5 Right) is characterised by the following steps:

1. The mask obtained from scheme two is used to create two sequences, adding the grey levels of the rows and columns, respectively. These two sequences provide four boundary points, which define two segments of different lengths in the horizontal and vertical dimensions.
2. Sequences of added grey levels in the vertical and horizontal dimensions of the mask are used to identify the RoI associated with the lungs, taking advantage of the higher added values outside the lungs.
3. A new mask is defined by identifying the rectangular template containing the RoI and blacking out the rest of the image. This mask is superimposed on the image.
4. A new equalisation is performed considering only those pixels with values different from zero.

The last two schemes are carried out to decrease the data’s variability, make the network training process simpler, and force the network to focus its attention on the region of the lungs, removing external characteristics that might influence the results obtained. For all three described pre-processing schemes, the images’ size was kept unchanged to guarantee the bounding boxes’ validity and the experiments’ comparability. However, considering that the bounding boxes cover areas outside the lungs for a large number of images in the data set, the size of the bounding boxes was resized by removing any part outside the rectangular template containing RoI associated with the lungs.

All models for each of the pre-processing schemes were trained using bounding boxes that were resized to the RoI that contains the lungs. Nevertheless, for the sake of comparison, the models were also evaluated using the size of the original bounding boxes. Moreover, a correction factor was applied to the results obtained with the original bounding boxes, to compensate for the IoU loss due to the resizing of the bounding boxes. These three evaluation procedures are named in the results Section 3: original, corrected, and resized bounding boxes. The correction factor was defined as the average IoU between the original and the resized bounding boxes. The global correction factor was estimated as 0.94, even though a particular correction factor was calculated and applied independently for each fold, considering only the samples used for training in every fold.

### 2.3. Data Augmentation

Data augmentation is a well-known technique to improve the performance of Machine Learning models [32]. It helps the model to generalise better by performing transformations of the original data. The following transformations were applied:

- Rotation of the image with a limit of 10º and with a probability of 0.25;
- Horizontal flip of the image with a probability of 0.5;
- A random brightness and contrast change with a probability of 0.2;
- One of the following transformations per sample:
  Motion blur with a probability of 0.2;
  Median blur with a probability of 0.1 and limited blurriness;
  Standard blur with a probability of 0.1 and limited blurriness.

### 2.4. Modelling

Object detection is a very active area of research, and as such, new models are released every year, bringing new architectures and techniques, often surpassing the previous models in accuracy and/or speed.

Almost all object detectors developed throughout the years fall into two main categories: one-stage detectors and two-stage detectors, the latter being the first one to appear. The main difference between these two architectures is that in two-stage algorithms, a region proposal system is used to first propose an RoI prior to the proper object detection process. In general terms, this leads to better-quality predictions at the cost of processing time. On the other hand, one-stage detectors skip this process by directly using the input image ‘as is’, reducing processing time and leading to faster detectors, usually at the cost of accuracy. Ever since their appearance, these two types of object detectors have evolved differently over the years, taking advantage of the appearance of CNN in computer vision. In addition to one- and two-stage object detectors, a third novel architecture has emerged in the last few years based on the new transformer deep learning models.

In this work, several deep learning-based object detection models are tested and later ensembled to improve predictions. The choice of the models is strongly motivated by their performance in previous works and their hardware requirements for training. The models developed are grounded on architectures based on Faster R-CNN, Cascade R-CNN, RetinaNet, and YoloV5.

All models are fine-tuned from pre-trained weights obtained using ImageNet [33] and COCO [34] data sets. Although these data sets contain images that are far different from the CXR images, the experiments have shown that the accuracy obtained improves and the training converges more easily than training the models from scratch. The criteria used to choose the selected models are that their performance must have been widely tested before, that their training requirements in terms of memory and graphics processing unit (GPU) are reasonable, and finally that the inner workings of the model are well-documented and well-understood.

A brief description of these architectures and the specific configuration is presented in the following subsections.

#### 2.4.1. Faster R-CNN

Faster R-CNN is one of the most well-known two-stage object detection algorithms. In this architecture, a CNN backbone extracts features from the proposed regions of the input image and the feature maps are fed into an RoI pooling layer. This layer performs a pooling operation, creating a fixed-size feature map, which is later fed to several fully connected layers (FC). Finally, the network contains two heads, one with a *softmax* activation function that performs classification, and the other that calculates the regression of the bounding box using the parameterisation of its coordinates. Faster R-CNN introduces a Region Proposal Network (RPN), which is trained to extract the candidate regions for the detection of objects. To do so, it employs a sliding window and a system of translation-invariant anchors. The anchors are centred on the sliding window and have different scales and shapes, so they can effectively detect objects of different sizes. Typically, nine different anchors are used, and the regression head is trained to learn the transformations of these anchors into the final bounding boxes. This network is trained using a multitask loss, which addresses both the regression and the classification problems. There are two stages in which the bounding boxes are predicted in an almost similar way: after the RPN and after the detection head.

Two different backbones are tested for this model: ResNet50 and ResNeXt101. The first stage of this backbone is frozen during the training phase, as it often does not provide substantial increases in accuracy, but results in a longer and more unstable training.

The developed model uses channels of size {256, 512, 1024, 2048} for the FPN section; anchors of ratio {0.5, 1, 2}; and strides of {4, 8, 16, 32, 64} pixels, which is the default setting commonly used in the literature. This configuration has demonstrated the ability to detect objects of different sizes in the image. Bounding box distance vectors are normalised to have zero mean and standard deviation {0.1, 0.1, 0.2, 0.2} and the images are normalised to have a target mean {123.675, 116.28, 103.53} and a standard deviation {58.395, 57.12, 57.375}.

The second stage of the model is modified so that the number of predicted classes is equal to 1, which corresponds to a lung opacity, plus an additional background class. The model is trained using the Cross-Entropy Loss function for classification and the robust L1 loss for bounding box regression.

#### 2.4.2. Cascade R-CNN

Cascade R-CNN is mainly based on Faster R-CNN but it aims to solve one of its key problems. In its release paper [35], the authors showed that in most cases, an object detector could only be optimised for a single IoU threshold value (this is 0.5, 0.6, 0.7, etc.). Therefore, a detector with a series of consecutive stages was proposed, where each stage is optimised for a single IoU threshold. Loss functions are identical to the ones proposed for Faster R-CNN and the key point in this architecture is that each bounding box regressor is optimised for the distribution of the previous bounding box regressor rather than for the original bounding box distribution.

A Cascade R-CNN model [35] with 3 stages is implemented with ResNeXt101 as its backbone, freezing its first stage. Positive detection thresholds are set to an IoU {0.5, 0.6, 0.7}. A FPN is used with channels of size {256, 512, 1024, 2048}, anchors with ratio {0.5, 1, 2}, and strides of {4, 8, 16, 32}. Images are normalised, as in the Faster R-CNN case. Bounding box distance vectors are normalised to have zero mean and standard deviation {0.1, 0.1, 0.2, 0.2} and are divided by the number of stages so that the standard deviation decreases between stages. The number of classes is set to 1 plus an additional background class. Cascade R-CNN is trained for using independent loss functions for each stage (Cross-Entropy Loss is used for the classification task and L1 Loss is used for bounding box regression).

#### 2.4.3. RetinaNet

RetinaNet [36] is a one-stage detector designed to perform with a precision comparable to that of two-stage detectors. The authors of RetinaNet noticed that one of the biggest problems with one-stage detectors was the extreme class imbalance between background class and foreground class due to the fact that one-stage detectors do not employ an RPN. To tackle this problem, a new loss function was introduced: the Focal Loss. This is a variant of the Cross-Entropy Loss where a weight factor is introduced to address the class imbalance. This term reduces the loss for well-classified samples and increases it for wrongly classified samples. In practise, this means that the network focuses on classifying the harder samples rather than the easy ones. RetinaNet uses a CNN backbone to extract the features of the image. The concept of anchors was ’borrowed’ from two-stage detectors. In RetinaNet anchors with areas from 322 to 5122 and ratios 1:1, 1:2, and 2:1 are used. Additionally, RetinaNet incorporates an FPN, being one of the first models to take advantage of this design in the literature. The model has three identical output heads, and each one is optimised for object scale detection across the image using the features of the corresponding FPN level.

A RetinaNet [36] model with a ResNext101 as its backbone was used, followed by an FPN with channels of sizes {256, 512, 1024, 2048}. The anchors used have a ratio {0.5, 1, 2} and strides of {4, 8, 16, 32}. Bounding box distance vectors are normalised to have zero mean and standard deviation {0.1, 0.1, 0.2, 0.2}. Bounding box distance vectors are normalised to have zero mean and unit standard deviation. Focal Loss is used as the classification loss with parameters γ = 2, α = 0.25 and L1 Loss as the bounding box regression loss.

#### 2.4.4. YoloV5

YoloV5 is a one-stage object detection model that has been built upon all of its previous versions. It is a model that is still in the development phase, and since its first appearance in 2020, it has received multiple upgrades and architectural changes. There are three distinctive parts in the YoloV5 structure: backbone, neck and detection head. The backbone of YoloV5 is based on the Cross Stage Partial Networks technique [37] in which a copy of the feature map of the dense layer is sent to the next stage of the network. The rationale behind this processing is to increase feature propagation so that information is not lost after the convolutions, and also to mitigate problems related to vanishing gradients.

For the neck of this object detection model, a PA-Net is used. It works in a similar way to the FPN of the RetinaNet model, and it is used to generate a pyramid of features. As previously, object detection is performed on different scales. Finally, a detection head uses the features obtained from the neck to predict the bounding boxes and their corresponding scores. The YoloV5 uses 3 different output scales to predict different scale objects in a given image. The largest convolutional output is responsible for detecting the largest scale object and vice versa.

In YoloV5 models, anchor box priors are used to predict bounding boxes in every image. The choice of these priors is made using a genetic algorithm to evolve the anchors into an optimal size and scale using recall as the metric to optimise.

The version of YoloV5 used in this work is the 6.0 release. YoloV5-6 is chosen because it is the largest YoloV5 model that fits into the memory of the GPU used. The backbone is a variation of CSPDarknet CNN in which the depth (number of layers) and width (number of convolutional filters) are controlled by two hyperparameters: depth-multiple, which is set to 1.33, and width-multiple, which is set to 1.25 respectively. In the neck of the object detector, a 2-level PA-Net is used. The model is trained with 3 warm-up epochs using the Complete IoU (CIoU) Loss function.

#### 2.4.5. Models Ensemble

**Figure 6:**
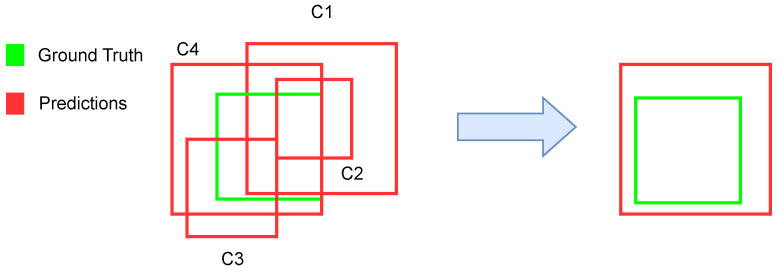
Schematic view of the Weighted Box Fusion algorithm. In this case $C_{1}>C_{2}>C_{3}>C_{4}$.

A Weighted Box Fusion [19] technique is used to combine the information given by the aforementioned architectures. This technique weights the predictions of the different models $C_{i}$ to produce new box coordinates and confidence scores as shown in Figure 6.

#### 2.4.6. Semi-Supervised Learning Using Pseudo-Labels

Aiming to improve the detector’s performance, a semi-supervised learning strategy was carried out using pseudo-labels, which consists of a two-stage training process.

Initially, the models are trained and validated during the first stage using the FISABIO-RSNA COVID-19 Detection data set described in Section 2.1.1, which contains labelled samples. The trained models are used to label samples from the HM Hospitales COVID-19 data set (described in Section 2.1.2), which is unlabelled. Those samples where the model predicted the label with high confidence are added to the initial data set and used for a second training round. This methodology was originally proposed in [25] to solve a conventional classification problem, and it was given the name of co-training, but currently, it is popularized as pseudo-labels training, although there are also self-supervised strategies based on clustering that use the same denomination [38].

The confidence score to select pseudo-labels was fixed to 0.7 for all architectures tested according to thresholds set in previous works [18]. As commented before, this technique can generate many incorrect pseudo-labels leading to noisy training due to calibration issues of the different architectures, and to the fact that classification scores are not strongly correlated with the precision of box localisation [28]. Moreover, the performance of different detectors might differ for the same threshold, making them difficult to compare. Current approaches to dealing with noisy labels rely on consistency regularisation-based methods, which have achieved strong performance in semi-supervised settings. However, they rely heavily on domain-specific data augmentations, which are not trivial to generate for every context [39]. Since a tailored selection of confidence thresholds per model is unfeasible, in this work pseudo-labels obtained by evaluating the best model (according to the first training stage) were used to re-train all the models and their ensembles, so that comparability among the different schemes is guaranteed.

## 3. Results

The training was conducted following a cross-validation procedure with 5 folds, so data were split 8/2 for training/validation. Several experiments were conducted to determine the number of epochs for the model to converge nicely. The final number of epochs was fixed at 80 and a Stochastic Gradient Descent (SGD) optimiser with a momentum of 0.9 was chosen for all models. Cosine Annealing *lr* schedule was used with a linear warm-up, a minimum *lr* of $10^{-7}$, and a maximum of $10^{-4}$ for all architectures, except for YoloV5, which used 0.002 and 0.2.

**Figure 7:**
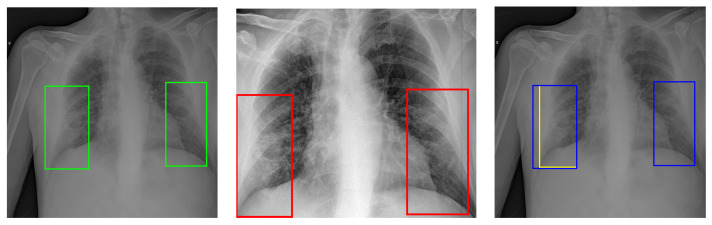
Bounding boxes before and after the cropping procedure of the images. (**Left**): Original image with its original bounding boxes (green line). (**Centre**): Cropped image with resized bounding boxes (red line). The left original bounding box falls partly outside the area cropped. As a result, its size is resized. (**Right**): Comparison of the original (blue line) and resized (yellow line) bounding boxes.

The models developed are grounded on the architectures mentioned above, based on Faster R-CNN, Cascade R-CNN, RetinaNet, and YoloV5. For each architecture, three experiments were carried out according to the pre-processing schemes presented in Section 2.2.1, namely: equalisation; equalisation + cropping; and equalisation + semantic lung segmentation. Furthermore, results were calculated for different evaluation procedures of the bounding boxes: original (Org) (Figure 7 Left), corrected (Corr), and resized (Resz) (Figure 7 Centre and Right).

Table 2 presents the results in terms of their mean (μ) mAP@50 ± its standard deviation (σ) for all combinations of architectures, pre-processing methods, and evaluation procedures. These results were used as a baseline for a further comparison following other approaches based on ensembles and/or using a semi-supervised training strategy. The results show the best performance for Cascade R-CNN and RetinaNet, YoloV5 being the worst.

Table 3 shows the results of the paired ensembles of the different architectures used. Ensembles of three or more architectures are not presented because the results did not provide significant improvements. The combination of RetinaNet and Cascade R-CNN provided the best results, improving the baseline presented in Table 2. This is consistent with the results provided in Table 2, which report that these two architectures are those with the best performance.

In addition, Table 4 shows, for the three architectures used and for the three pre-processing schemes, the number of samples from the HM Hospitales Data Set that reported a confidence score > 0.7 after training/validation of the models. These results were used to identify the best scheme to be used for further semi-supervised training. As expected, results significantly depend on the architecture, Faster CNN and Cascade R-CNN which provide more candidate samples to be used as pseudo-labels. In view of these results, Cascade R-CNN was preferred due to its best results reported in Table 2. It is worth mentioning the strong differences among the models in terms of the number of bounding boxes detected with scores > 0.7. A particularly striking fact is that, according to Table 2, the performance of Cascade R-CNN and RetinaNet is quite comparable, but the number of pseudo-labels obtained by each model is completely different for the same confidence level, showing RetinaNet a clear under-confident behaviour and evidencing calibration issues of these models.

On the other hand, Table 5 shows again, for all architectures, pre-processing methods and evaluation procedures the results using the semi-supervised approach. Performance increased significantly in all cases, with RetinaNet being the best architecture.

Finally, Table 6 shows again, that the ensemble of RetinaNet and Cascade R-CNN in combination with the semi-supervised method provides the best performance of all experiments carried out, providing a significant improvement. This is consistent with the results provided in Table 5, which report that these two architectures are the best at detecting lung opacities associated with COVID-19.

**Figure 8:**
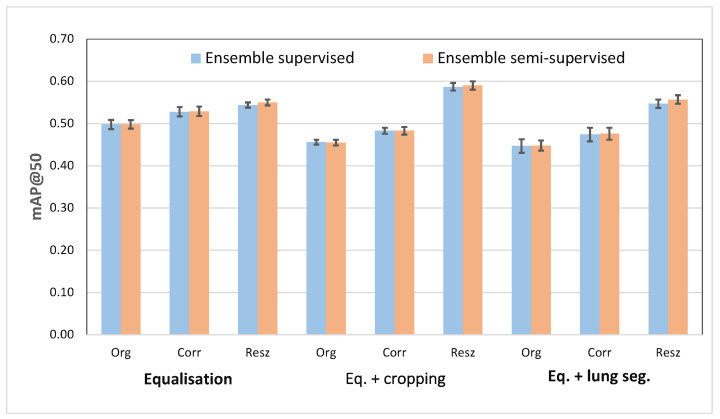
Best results for the ensembles with RetinaNet and Cascade R-CNN.

The main results are summarised in Figure 8, which graphically shows the most significant outcomes obtained for the ensembles of Cascade R-CNN and RetinaNet, also comparing the results using supervised and semi-supervised approaches. In numerical terms, the best scheme achieved a mAP@50 of 0.59.

A common result in all experiments carried out is that no matter the architecture (Faster R-CNN, Cascade R-CNN, RetinaNet, YoloV5), the evaluation procedure (Original, Corrected, Resized) the training method (supervised or semi-supervised), or the ensembles used, the second pre-processing method (equalisation + cropping) always boosted the performance of the system, suggesting that forcing the attention on the RoI of the lungs makes the identification process easier, whereas results are very similar for the first and third pre-processing schemes (equalisation, and equalisation + lung segmentation).

## 4. Discussion and Conclusions

The present work addressed the task of automatically identifying lung opacities due to COVID-19 from CXR using the FISABIO-RSNA COVID-19 Detection open data set [16]. In this respect, the system generates the coordinates of the bounding boxes where the opacities are found, adjusting their areas to the regions where these opacities are present. This is carried out using techniques inherited from the object detection field, which have demonstrated good performance for other tasks. The results report that the opacities due to COVID-19 can be detected with a mAP@50 of 0.59 following a semi-supervised training procedure and an ensemble of two architectures: RetinaNet and Cascade R-CNN.

The paper contributes to the detection of lung lesions due to COVID-19 from CXR images with additional results. Contributions are mainly in a comprehensive presentation of the results (and their systematic comparison), and in the effect of different pre-processing strategies applied to the images. The goal of the pre-processing techniques used is to guide the attention of the model by reducing the search space, and also reducing artefacts that could confuse the networks (such as the burned-in meta information that usually appears at the top/bottom of the images, including patient’s info, data about the recording procedure, etc.). In this regard, this article explores the effect of cropping radiological images on the rectangular region covered by the lungs, and their semantic segmentation.

For this purpose, the paper presents results using four different off-the-shelf ANNs widely used in the field of object detection: Faster R-CNN, Cascade R-CNN; RetinaNet, and YoloV5. In addition, different combinations of ensembles of these models were also fused using a WBF approach, following both supervised and semi-supervised learning schemes. Results are compared using the mAP@50 metric averaged after a five-fold cross-validation. The selection of these simple architectures stands on their wide use, so they represent a good framework for comparing the pre-processing methodologies proposed. It is worth noting that the aim of this work is neither the development of new architectures nor the improvement of the accuracy with respect to the state of the art, but also comparing and discussing more interpretable approaches through the aforementioned pre-processing strategies.

The results obtained are thoughtfully and systematically presented, so comparisons among the different approaches are straight, contributing to a fair comparison of the different schemes developed, and establishing a methodological framework which can also be extrapolated to other architectures, ensembles and/or procedures for the same purpose.

In general terms, the best results were obtained using Cascade R-CNN and RetinaNet. Furthermore, results showed that paired ensembles improve the performance, being the pair formed by RetinaNet and Cascade R-CNN the best combination of those tested. The gain by using three or more architectures was not relevant.

On the other hand, as expected, semi-supervised training using the pseudo-labels provided by Cascade R-CNN also improves the performance, but lightly.

Cascade R-CNN and RetinaNet were consistent throughout the entire experimental phase. However, their performance in labelling samples for the semi-supervised approach was drastically different, showing RetinaNet an under-confident behaviour and casting doubts about its generalisation capabilities out of the training data set. Regarding pseudo-labels identification, RetinaNet found lung opacities with sufficient confidence in only a few hundred out of more than five thousand samples and, in most cases, bounding boxes were detected for only one of the lungs. Cascade R-CNN provided more consistent results for this task, so it was preferred for this task.

In any case, it is worth noting that semi-supervised training was carried out using those images that reported a confidence score over 0.7 for all models. This threshold leads to significant differences in the architectures used, due to the poor calibration of the models, which provide scores that cannot be compared, even when accuracies are similar and scores could be interpreted as probabilities. On the other hand, the scores provided are related to the estimation of the category associated with the object, but classification scores are not necessarily correlated with the precision of box location [28].

To interpret the results, it is worth remembering that the experiments were evaluated following three evaluation procedures (original, corrected, and resized). Of these three procedures, the third is the one providing the most realistic evaluation scenario, since it takes into account the resizing of the bounding boxes, having removed those regions of them falling outside the rectangular RoI of the lungs (which are considered not relevant for our purpose). This evaluation procedure is considered more accurate even in the first pre-processing scenario, since it improves the quality of the bounding boxes, removing noisy information from them.

A typical result in all experiments carried out is that no matter the architecture, the training method, or the ensemble used, and according to the aforementioned resized evaluation procedure, the cropping to the rectangular RoI occupied by the lungs always improved the performance of the system. This result suggests that forcing the attention on such RoI makes the identification process easier, but also suggests the need of resizing the available bounding boxes used to delineate the opacities according to the information provided by the cropping procedure itself. This process, which is carried out automatically, helps to remove noise and inaccuracies in the labelling procedure, also leading to results that are considered more accurate.

On the other hand, semantic segmentation of the lungs has provided improvements with respect to the baseline based on raw images, but not significant. This is mainly attributed to errors in semantic segmentation, which sometimes remove significant areas, especially in the most peripheral part of the lungs, where opacities are more common [13]. This is also attributed to oversized bounding boxes, which are correctly placed over the opacities, but with a much larger area over the mask.

By visually assessing the model’s predictions it can be seen that the model predicts, most of the time, a unique bounding box around the affected lung, which matches closely with the ground truth annotation. This is mainly due to the way the boundaries were drawn by the radiologists, who enclosed several individual lesions into a single larger bounding box, which makes the bounding box areas overly estimated. This poses a limitation in the effectiveness of the system, as it seems that often the models try to predict a bounding box around the entire lung instead of focussing on the smaller areas of the lung with opacities.

Overall, the system built in this work achieves results (in terms of mAP@50) which are comparable (or better) to those obtained in the state of the art, although a direct comparison with them is not straightforward: even when the data set used for training is the same (SIIM-FISABIO-RSNA COVID-19 Detection Challenge [16]), the validation data are different since they were not made available by the challenge organisers.

The limited accuracy of the results (mAP@50 = 0.59) is mainly a consequence attributed to the limited accuracy of the bounding boxes provided in the data set. However, there are also limitations in the architectures used, which were specifically developed to detect well-defined objects, not textures with undefined shapes (and/or boundaries) like those corresponding to the opacities.

In any case, this research has several limitations, mainly related to the data set used and to the inherent characteristics of the ANNs: (i) The first is grounded on the labels used for training (i.e., the bounding boxes). The FISABIO-RSNA COVID-19 Detection Data Set is widely used because it is freely available, but the bounding boxes provided (even when they identify the opacities well) are not very specific, since they were delineated to pick up several opacities inside (instead of using several bounding boxes, one for each opacity). In addition, many bounding boxes have a certain area that falls outside the rectangular RoI covered by the lungs, introducing noise in the training phase and reducing the performance of the automatic system. In this regard, this paper provides a method to lightly mitigate this effect. (ii) Another limitation is related to the calibration of the models, which affects the behaviour of the semi-supervised approach. Recent strategies to calibrate networks and evaluate score uncertainties are required to get more accurate pseudo-labels.

## Figures and Tables

**Table 2:** Performance of the different model architectures for different image pre-processing strategies and evaluation procedures. The BBox column indicates the evaluation procedure for the bounding boxes used during validation: Originals (Org), Corrected (Corr), Resized (Resz).

		Model Architecture			
**Processing**	**BBox**	**Faster**	**Cascade**	**RetinaNet**	**YoloV** **5**
	Org	0.407 ± 0.013 *	0.465 ± 0.008	0.478 ± 0.013	0.379 ± 0.019
Equalisation	Corr	0.432 ± 0.015	0.493 ± 0.010	0.507 ± 0.014	0.402 ± 0.020
	Resz	0.434 ± 0.006	0.525 ± 0.007	0.508 ± 0.010	0.435 ± 0.018
	Org	0.386 ± 0.013	0.439 ± 0.006	0.428 ± 0.007	0.370 ± 0.014
Equalisation + cropping	Corr	0.410 ± 0.015	0.465 ± 0.005	0.454 ± 0.008	0.392 ± 0.016
	Resz	0.482 ± 0.011	0.569 ± 0.007	0.555 ± 0.008	0.476 ± 0.020
	Org	0.348 ± 0.015	0.430 ± 0.017	0.417 ± 0.012	0.317 ± 0.027
Equalisation + lung segmentation	Corr	0.369 ± 0.015	0.456 ± 0.017	0.443 ± 0.013	0.336 ± 0.027
	Resz	0.426 ± 0.015	0.533 ± 0.010	0.505 ± 0.014	0.409 ± 0.023

* μmAP@50 ± σ.

**Table 3:** Performance of the different model ensembles for different image pre-processing strategies and evaluation procedures. The BBox column has the same meaning as in Table 2.

		Model Architecture			
**Processing**	**BBox**	**RetinaNet &**	**RetinaNet &**	**Cascade &**	**Cascade &**
		**Cascade**	**Faster**	**Faster**	**YoloV** **5**
	Org	0.498 ± 0.011 *	0.476 ± 0.014	0.471 ± 0.009	0.472 ± 0.008
Equalisation	Corr	0.528 ± 0.011	0.506 ± 0.016	0.500 ± 0.010	0.501 ± 0.010
	Resz	0.544 ± 0.006	0.503 ± 0.008	0.523 ± 0.008	0.527 ± 0.006
	Org	0.456 ± 0.006	0.432 ± 0.008	0.442 ± 0.008	0.446 ± 0.005
Equalisation + cropping	Corr	0.483 ± 0.007	0.458 ± 0.008	0.469 ± 0.009	0.473 ± 0.006
	Resz	0.587 ± 0.009	0.548 ± 0.009	0.561 ± 0.008	0.571 ± 0.007
	Org	0.447 ± 0.016	0.413 ± 0.016	0.432 ± 0.016	0.435 ± 0.012
Equalisation + lung segmentation	Corr	0.474 ± 0.016	0.438 ± 0.016	0.459 ± 0.016	0.462 ± 0.012
	Resz	0.547 ± 0.010	0.497 ± 0.014	0.527 ± 0.013	0.536 ± 0.010

* μmAP@50 ± σ.

**Table 4:** Number of samples with detected bounding boxes (pseudo-labels) per fold on the HM Hospitales data set.

		Samples per Fold				
**Processing**	**Model**	**1**	**2**	**3**	**4**	**5**
Equalisation	Faster	4421	3929	4020	4125	4043
	Cascade	4135	3807	4032	3862	3809
	RetinaNet	124	178	122	114	100
	YoloV5	0	0	0	0	0
Equalisation + cropping	Faster	4140	4170	4047	4061	4207
	Cascade	4275	3967	3855	3949	4118
	RetinaNet	249	272	250	257	235
	YoloV5	0	8	0	33	19
Equalisation + lung segmentation	Faster	3995	3891	3887	4137	4116
	Cascade	4171	3734	3823	4133	3947
	RetinaNet	34	34	22	46	35
	YoloV5	0	1	0	0	0

**Table 5:** Performance of the different model architectures for different image pre-processing strategies, evaluation procedures, and a semi-supervised approach. The BBox column has the same meaning as in Table 2.

		Model Architecture			
**Processing**	**BBox**	**Faster**	**Cascade**	**RetinaNet**	**YoloV5**
	Org	0.439 ± 0.013 *	0.445 ± 0.011	0.486 ± 0.009	0.339 ± 0.024
Equalisation	Corr	0.466 ± 0.014	0.472 ± 0.013	0.516 ± 0.011	0.360 ± 0.025
	Resz	0.495 ± 0.009	0.518 ± 0.014	0.540 ± 0.004	0.428 ± 0.022
	Org	0.407 ± 0.016	0.431 ± 0.006	0.432 ± 0.010	0.385 ± 0.023
Equalisation + cropping	Corr	0.431 ± 0.017	0.457 ± 0.007	0.458 ± 0.011	0.409 ± 0.024
	Resz	0.523 ± 0.016	0.562 ± 0.010	0.577 ± 0.013	0.483 ± 0.013
	Org	0.398 ± 0.019	0.409 ± 0.016	0.437 ± 0.019	0.316 ± 0.028
Equalisation + lung segmentation	Corr	0.422 ± 0.019	0.434 ± 0.017	0.464 ± 0.020	0.335 ± 0.029
	Resz	0.494 ± 0.016	0.515 ± 0.021	0.552 ± 0.010	0.412 ± 0.019

* μmAP@50 ± σ.

**Table 6:** Performance of the different model ensembles for different pre-processing strategies, evaluation procedures and a semi-supervised approach. The BBox column has the same meaning as in Table 2.

		Model Architecture			
**Processing**	**BBox**	**RetinaNet &**	**RetinaNet &**	**Cascade &**	**Cascade &**
		**Cascade**	**Faster**	**Faster**	**YoloV5**
	Org	0.498 ± 0.010 *	0.490 ± 0.010	0.458 ± 0.013	0.459 ± 0.007
Equalisation	Corr	0.529 ± 0.011	0.520 ± 0.012	0.486 ± 0.015	0.487 ± 0.009
	Resz	0.550 ± 0.007	0.542 ± 0.006	0.524 ± 0.011	0.534 ± 0.010
	Org	0.455 ± 0.007	0.440 ± 0.010	0.438 ± 0.008	0.445 ± 0.003
Equalisation + cropping	Corr	0.483 ± 0.009	0.467 ± 0.011	0.464 ± 0.010	0.473 ± 0.004
	Resz	0.590 ± 0.010	0.576 ± 0.012	0.565 ± 0.009	0.569 ± 0.010
	Org	0.448 ± 0.012	0.442 ± 0.018	0.418 ± 0.013	0.426 ± 0.012
Equalisation + lung segmentation	Corr	0.476 ± 0.014	0.469 ± 0.018	0.444 ± 0.014	0.452 ± 0.013
	Resz	0.557 ± 0.010	0.550 ± 0.008	0.522 ± 0.015	0.530 ± 0.014

* μmAP@50 ± σ.

## Data Availability

The models used in this work were trained using the SIIM-FISABIO-RSNA COVID-19 Detection data set [17] and the HM Hospitales data set [31].

## Abbreviations

The following abbreviations are used in this manuscript:

AI	Artificial Intelligence
AP	Anterior-posterior
CIoU	Complete Intersection over Union
CLAHE	Contrast Limited Adaptive Histogram Equalisation
CXR	Plain Chest X-ray
CT	Computer Tomography
CR	Computed Radiography
DSS	Decision Support System
DX	Digital Radiography
GPU	Graphics Processing Unit
HSV	Hue, Saturation, Lightness
Faster-R-CNN-FPN	Faster Region-Based Convolutional Neural Network Feature
	Pyramid Network
FC	Fully connected layers
FISABIO	Foundation for the Promotion of Health and Biomedical Research of
	Valencia Region
FPN	Feature Pyramid Network
HIPAA	Health Insurance Portability and Accountability Act
IoU	Intersection over Union
*lr*	Learning rate
mAP@50	Mean Average Precision with an IoU of at least 0.5
MERS	Middle East Respiratory Syndrome
NMW	Non-Maximun Weighted
SARS	Severe Acute Respiratory Syndrome
SGD	Stochastic Gradient Descent
PA	Posterior-anterior
R0	Reproduction number
R-CNN	Region-based Convolution Neural Network
RICORD	RSNA International COVID-19 Open Radiology Database
RoI	Region of Interest
RNA	Ribonucleic Acid
RPN	Region Proposal Network
RSNA	Radiological Society of North America
SGD	Stochastic Gradient Descent
SIIM	Society for Imaging Informatics in Medicine
TTA	Test Time Augmentation
WBF	Weighted Box Fusion
Yolo	You Only Look Once
